# Risks of metabolic syndrome in the ADVANCE and NAMSAL trials

**DOI:** 10.3389/frph.2023.1133556

**Published:** 2023-09-18

**Authors:** Tamara Tovar Sanchez, Mireille Mpoudi-Etame, Charles Kouanfack, Eric Delaporte, Alexandra Calmy, Francois Venter, Simiso Sokhela, Bronwyn Bosch, Godspower Akpomiemie, Angela Tembo, Toby Pepperrell, Bryony Simmons, Carmen Perez Casas, Kaitlyn McCann, Manya Mirchandani, Andrew Hill

**Affiliations:** ^1^ TransVIHMI, University of Montpellier, IRD, INSERMI, Montpellier, France; ^2^ Infectious Diseases, Regional Military Hospital Number 1, Yaoundé, Cameroon; ^3^ Faculty of Medicine and Pharmaceutical Sciences, University of Dschang, Dschang, Cameroon; ^4^ Day Stay Hospital, Central Hospital of Yaoundé, Henri-Dunant, Yaoundé, Cameroon; ^5^ ANRS Cameroon Site, Central Hospital of Yaoundé, Henri-Dunant, Yaoundé, Cameroon; ^6^ Division of Infectious Diseases, HIV-AIDS Unit, Genva University Hospitals, Geneva, Switzerland; ^7^ Faculty of Health Sciences, University of the Witwatersrand, Johannesburg, South Africa; ^8^ School of Medicine and Veterinary Medicine, University of Edinburgh, Edinburgh, United Kingdom; ^9^ London School of Economics and Political Science, LSE Health, London, United Kingdom; ^10^ Unitaid, Global Health Campus, Le Grand-Saconnex, Switzerland; ^11^ Faculty of Medicine, Imperial College London, London, United Kingdom; ^12^ Department of Pharmacology and Therapeutics, University of Liverpool, Liverpool, United Kingdom

**Keywords:** metabolic syndrome, cardiovascular, antriretroviral medication, tenoforvir disoproxil fumarate, tenofovir alafenamide

## Abstract

**Introduction:**

The ADVANCE and NAMSAL trials evaluating antiretroviral drugs have both reported substantial levels of clinical obesity in participants. As one of the main risk factors for metabolic syndrome, growing rates of obesity may drive metabolic syndrome development. This study aims to evaluate the risk of metabolic syndrome in the ADVANCE and NAMSAL trials.

**Methods:**

The number of participants with metabolic syndrome was calculated at baseline and week 192 as central obesity and any of the following two factors: raised triglycerides, reduced HDL-cholesterol, raised blood pressure and raised fasting glucose. Differences between the treatment arms were calculated using the *χ*^2^ test.

**Results:**

Across all visits to week 192, treatment-emergent metabolic syndrome was 15% (TAF/FTC + DTG), 10% (TDF/FTC + DTG) and 7% (TDF/FTC/EFV) in ADVANCE. The results were significantly higher in the TAF/FTC + DTG arm compared to the TDF/FTC/EFV arm (*p* < 0.001), and the TDF/FTC + DTG vs. the TDF/FTC/EFV arms (*p* < 0.05) in all patients, and in females. In NAMSAL, the incidence of treatment-emergent metabolic syndrome at any time point was 14% (TDF/3TC + DTG) and 5% (TDF/3TC + EFV) (*p* < 0.001). This incidence was significantly greater in the TDF/3TC/DTG arm compared to the TDF/3TC/EFV arm in all patients (*p* < 0.001), and in males (*p* < 0.001)

**Conclusion:**

In this analysis, we highlight treatment-emergent metabolic syndrome associated with dolutegravir, likely driven by obesity. Clinicians initiating or monitoring patients on INSTI-based ART must counsel for lifestyle optimisation to prevent these effects.

## Introduction

Metabolic syndrome is a cluster of clinical features comprising obesity, dyslipidaemia, hypertension, and impaired glucose tolerance. It has been defined by the International Diabetes Federation as having central obesity plus any two of the following: (1) raised triglycerides, (2) reduced high-density lipoprotein (HDL) cholesterol or on lipid lowering therapy, (3) hypertension, and (4) raised fasting plasma glucose or diagnosis of type 2 diabetes (1) (See Table 1). The mechanism of development is not fully understood, but has been linked to genetic predisposition, chronic inflammation, neurohormonal activation, and insulin resistance (2, 3). Visceral adiposity has been shown to be a significant trigger to activate pathways of metabolic syndrome development (2, 4).

**Table 1:** The new international diabetes federation (IDF) definition of metabolic syndrome.

According to the new IDF definition, for a person to be defined as having the metabolic syndrome they must have: Central obesity (defined as waist circumference^a^ with ethnicity specific values) plus, any two of the following four factors:	
Raised triglycerides	≥150 mg/dl (1.7 mmol/L) Or specific treatment for this lipid abnormality
Reduced HDL cholesterol	<40 mg/dl (1.03 mmol/L) in males <50 mg/dl (1.29 mmol/L) in females or specific treatment for this lipid abnormality
Raised blood pressure	Systolic BP ≥ 130 or diastolic BP ≥ 85 mm Hg or treatment of previously diagnosed hypertension
Raised fasting plasma glucose	(FPG) ≥100 mg/dl (5.6 mmol/L), or previously diagnosed type 2 diabetes If above 5.6 mmol/L or 100 mg/dl, OGTT is strongly recommended but is not necessary to define presence of the syndrome

^a^ If BMI is >30 kg/m^2^, central obesity can be assumed, and waist circumference does not need to be measured.

There has been a suggestion that metabolic syndrome may be more prevalent in people living with HIV (PLWH) in a meta-analysis conducted in Sub Saharan Africa, with a prevalence of 21.5% in PLWH compared to 12% in uninfected populations (5). The global prevalence has been estimated to be between 16.7% and 31.3% among PLWH in another study (6).

It has been widely reported that metabolic syndrome predisposes to development of cardiovascular disease and type II diabetes, with an estimated two and five-fold increased risk respectively, compared to those without the syndrome (1). It has also been linked to co-morbidities including cancer development, polycystic ovarian syndrome (7), obstructive sleep apnoea (8), chronic kidney disease (9), and non-alcoholic fatty liver disease (10, 11).

Previously, protease inhibitors used to treat HIV were associated with metabolic syndrome development (12). However, there is now a significant body of evidence associating weight gain and obesity with integrase inhibitors (INSTI) (13, 14), which are recommended as the preferred first line antiretroviral in the WHO guidelines for treatment of HIV-1 infection (15). Tenofovir alafenamide, often used in combination with INSTIs, has also been implicated in weight gain and obesity (13, 16). Weight gain associated with both INSTIs +/− TAF, has been greater in Black ethnicity, and females (13, 16). As one of the main risk factors for metabolic syndrome, growing rates of obesity within this patient population may drive metabolic syndrome development.

The NAMSAL-ANRS-12313 (NAMSAL) trial is a phase 3 open-label randomised trial which compared the efficacy of TDF/3TC + DTG to TDF/3TC + EFV (17). The trial recruited adults with HIV-1 infection who had not received antiretroviral therapy from three hospitals in Cameroon. The ADVANCE trial is a phase 3 randomised controlled open-label trial which evaluated the efficacy and safety of TAF/FTC + DTG and TDF/FTC + DTG, as compared with TDF/FTC/EFV (18). The trial recruited participants from routine HIV testing sites, based in Johannesburg, South Africa.

The ADVANCE and NAMSAL trials have both reported substantial levels of clinical obesity (17, 18). Weight gain has been more pronounced on the DTG arms of both trials, disproportionally affecting females, and particularly those on the TAF/FTC backbone on the ADVANCE trial (17, 18). This study aims to evaluate the risk of metabolic syndrome in the ADVANCE and NAMSAL trials.

## Methods

### Study design

The ADVANCE and NAMSAL trials were the first open-label, non-inferiority phase 3 randomised controlled trials of DTG with treatment-naïve participants in low- to middle-income country settings (LMIC), with recruitment of >99% Black Africans in both studies (17, 18). Ethics approval and written, informed consent for all participants were obtained and further details on these can be found in the original papers (17, 18). Study visits were planned at baseline, weeks 4, 12 and every 12 weeks thereafter until week 192. At each visit, data was recorded on parameters including HIV RNA, weight, systolic/diastolic blood pressure, fasting glucose, cholesterol, HDL, LDL and triglycerides. Both ADVANCE and NAMSAL ended at Week 192.

### Statistical analyses

Baseline characteristics including age, sex, weight, HIV RNA, CD4 count, and the metabolic parameter levels were calculated for each treatment arm in both the ADVANCE and NAMSAL trials. Count outcomes are summarised as number (%) and continuous outcomes as median (IQR). We also evaluated how many participants had abnormalities in any of the metabolic parameters (BMI, blood pressure, fasting glucose, triglycerides, HDL) at baseline and week 192. Results are displayed as number (%).

Metabolic syndrome at any time up to week 192 was calculated in both trials as central obesity (BMI > 30 kg/m^2^) and any of the following two factors: raised triglycerides (≥1.7 mmol/L), reduced HDL-cholesterol (<1.29 mmol/L), raised systolic or diastolic blood pressure (SBP ≥ 130 mm Hg or DBP ≥ 85 mm Hg) and raised fasting glucose (≥5.6 mmol/L), as per the definition set by the International Diabetes Federation (1) (Table 1). Participants who already had metabolic syndrome at baseline were excluded from the analysis. Results are displayed as number (%) of participants with metabolic syndrome.

Statistical analysis was conducted using STATA/IC version 16 (StataCorp LLC, College Station, TX, USA). Differences between the treatment arms were calculated using the *χ*^2^ test. The significance threshold was set at 5% (two-sided).

## Results

In the ADVANCE trial there were a total of 1,053 randomised participants (TAF/FTC + DTG = 351, TDF/FTC + DTG = 351, TDF/FTC + EFV = 351). The median age of participants was 32 in the TAF/FTC + DTG and TDF/FTC + DTG arms and 33 in the TDF/FTC + EFV arm. Females comprised 61% of participants in the TAF/FTC + DTG arm, 59% in the TDF/FTC + DTG arm and 57% in the TDF/FTC + EFV arm. The median weight of participants was 66.4 kg, 66.3 kg and 66.4 kg in the TAF/FTC + DTG, TDF/FTC + DTG and TDF/FTC + EFV arms respectively. In the NAMSAL trial there were a total of 616 randomised participants (TDF/3TC + DTG = 310, TDF/3TC + EFV = 303). The median age of participants was 38 in the TDF/3TC + DTG arm and 36 in the TDF/3TC + EFV arm. 63.5% of participants in the TDF/3TC + DTG arm and 68.3% in the TDF/3TC + EFV arm were females. The median weight of participants was 64 kg in both arms. Further details on baseline characteristics of participants in ADVANCE and NAMSAL are presented in Tables 2A,B.

**Table 2A:** Baseline characteristics: ADVANCE.

	TAF/FTC + DTG (*n* = 351)	TDF/FTC + DTG (*n* = 351)	TDF/FTC + EFV (*n* = 351)
Age	32 (27–38)	32 (26–37)	31 (27–37)
Female Sex	214 (61)	208 (59)	201 (57)
HIV RNA log_10_ copies/ml	4.43 (3.79–4.92)	4.40 (3.78–4.86)	4.36 (3.71–4.93)
CD4 count	320 (174–493)	275 (163–427)	297 (177–444)
Weight kg	66.4 (59.3–77.4)	66.3 (57.7–76.7)	66.4 (58.5–76.9)
Cholesterol mmol/L	3.75 (3.29–4.37)	3.66 (3.17–4.21)	3.72 (3.19–4.26)
HDL mmol/L	1.07 (0.87–1.33)	1.06 (0.89–1.3)	1.09 (0.9–1.32)
Glucose mmol/L	4.2 (3.9–4.5)	4.3 (3.9–4.6)	4.2 (3.8–4.6)
Triglycerides mmol/L	0.85 (0.66–1.19)	0.83 (0.65–1.09)	0.87 (0.67–1.19)
Systolic BP	120 (112–132)	121 (111–131)	120.5 (112–131)
Diastolic BP	79 (72–86)	80 (73–86)	79 (73–87)

Continuous values are displayed as Median (IQR) and count values as No. (%).

**Table 2B:** Baseline characteristics: NAMSAL.

	TDF/3TC + DTG (*n* = 310)	TDF/3TC + EFV (*n* = 303)
Age	38 (31–46)	36 (29–43)
Female Sex	197 (63.5)	209 (68.9)
HIV RNA log_10_ copies/ml	5.3 (4.8–5.8)	5.3 (4.7–5.8)
CD4 count per mm^3^	289 (157–452)	273 (147–428)
Weight kg	64 (58–73)	64 (56–71)
Cholesterol g/L	1.5 (1.3–1.7)	1.5 (1.3–1.8)
Triglycerides g/L	0.84 (0.61–1.08)	0.82 (0.61–1.08)
HDL g/L	0.4 (0.3–0.5)	0.4 (0.3–0.5)
Glucose g/L	0.82 (0.74–0.89)	0.81 (0.75, 0.88)
Systolic BP	115 (104–126)	114 (105–126)
Diastolic BP	72 (66–80)	71 (65–80)

## Advance

### Weight

**Figure 1:**
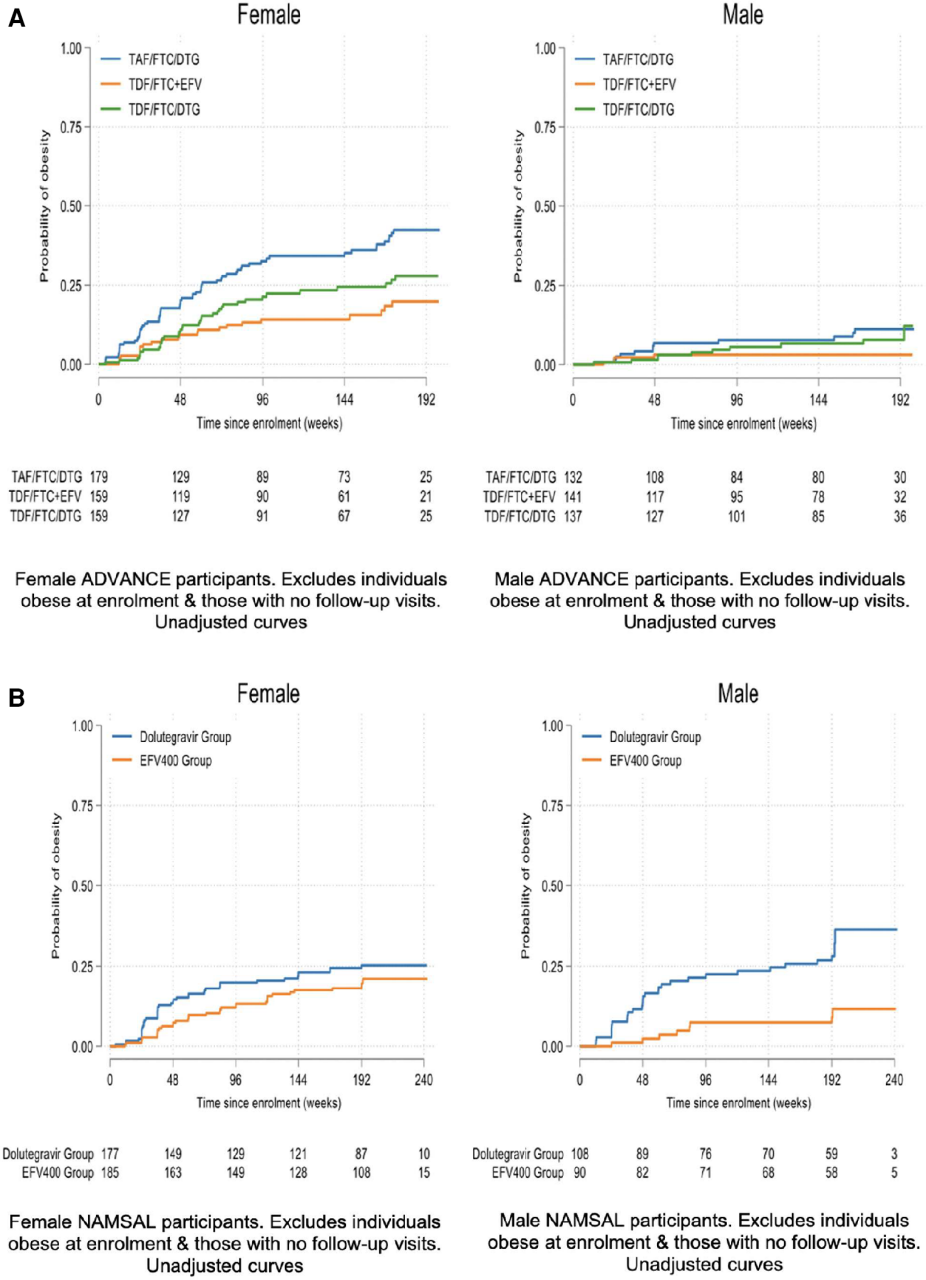
(**A**) Time to clinical obesity – ADVANCE. (**B**) Time to clinical obesity – NAMSAL.

At week 192, mean weight change across the TAF/FTC + DTG, TDF/FTC + DTG and TDF/FTC/EFV arms were 9.93 kg, 6.65 kg and 5.01 kg in females and 7.18 kg, 4.87 kg and 1.29 kg in males Figure 1A displays the time to clinical obesity across the 3 arms.

### Changes in metabolic parameters

In the ADVANCE trial, the only significant difference in metabolic abnormalities between treatment arms at baseline was for fasting glucose (*p* = 0.014). At week 192 there were significant differences between treatment arms for fasting glucose (*p* = 0.004), triglycerides (*p* = 0.017) and HDL (*p* < 0.00001). Table 3A shows the number (%) of participants with abnormalities in metabolic parameters stratified by treatment arm at baseline and week 192.

**Table 3A:** Abnormalities in metabolic parameters at baseline vs. week 192: ADVANCE.

	Group 1 (TAF/FTC + DTG) *n* (%)	Group 2 (TDF/FTC + DTG) *n* (%)	Group 3 (TDF/FTC/EFV) *n* (%)	*p* - *χ*^2^
BMI > 30				
Baseline	35/351 (10%)	48/351 (14%)	44/351 (13%)	0.30
Week 192	63/218 (29%)	53/202 (26%)	36/174 (21%)	0.17
SBP ≥ 130 or DBP ≥ 85				
Baseline	141/351 (40%)	136/351 (39%)	135/351 (38%)	0.88
Week 192	118/218 (54%)	108/202 (53%)	78/174 (45%)	0.14
Fasting glucose ≥5.6				
Baseline	2/348 (0%)	13/349 (4%)	7/351 (2%)	0.01
Week 192	8/218 (4%)	16/200 (8%)	22/174 (13%)	0.00
Triglycerides ≥1.7				
Baseline	27/348 (8%)	18/350 (5%)	30/351 (9%)	0.19
Week 192	28/218 (13%)	10/200 (5%)	20/174 (12%)	0.02
HDL < 1.29				
Baseline	251/348 (72%)	258/350 (71%)	258/351 (73%)	0.88
Week 192	142/218 (65%)	138/200 (69%)	75/173 (43%)	0.00

### Metabolic syndrome

51 (5%) participants had metabolic syndrome at baseline in the ADVANCE trial. There were no differences between the treatment arms. Across all study visits to week 192, treatment-emergent metabolic syndrome was 15% (TAF/FTC + DTG), 10% (TDF/FTC + DTG) and 7% (TDF/FTC/EFV) (Table 4A). The results were significantly higher in the TAF/FTC + DTG compared to the TDF/FTC/EFV arm (*p* < 0.001), and the TDF/FTC + DTG vs. the TDF/FTC/EFV arms (*p* < 0.05) in all patients, and in females. The results in males were non-significant. Table 4A shows the results stratified by study visit and sex. **NAMSAL**.

**Table 4A:** Metabolic syndrome at any time point in ADVANCE.

	TAF/FTC + DTG *n*/total (%)	TDF/FTC + DTG *n*/total (%)	TDF/FTC/EFV *n*/total (%)	TAF/FTC + DTG vs. TDF/FTC/EFV	TAF/FTC + DTG vs. TDF/FTC + DTG	TDF/FTC + DTG vs. TDF/FTC/EFV
All participants	50/335 (15)	32/330 (10)	23/337 (7)	0.00	0.21	0.03
Female	40/199 (20)	23/189 (12)	19/191 (10)	0.01	0.59	0.03
Male	10/136 (7)	9/141 (6)	4/146 (3)	0.07	0.13	0.74

### Weight

At week 192, mean weight change across the TDF/3TC + DTG and TDF/3TC + EFV arms were 6.37 kg and 5.38 kg in females and 5.13 kg and 3.51 kg in males. Figure 1B displays the time to clinical obesity across the 3 arms.

### Changes in metabolic parameters

In the NAMSAL trial, there was no significant difference in metabolic abnormalities between treatment arms at baseline (Table 3B). At week 192 there was a significant difference between treatment arms for BMI (*p* < 0.01), raised blood pressure (*p* < 0.01), and HDL (*p* < 0.05). Table 3B shows the number (%) of participants with abnormalities in metabolic parameters stratified by treatment arm at baseline and week 192.

**Table 3B:** Abnormalities in metabolic parameters at baseline vs. week 192: NAMSAL.

	TDF/FTC + DTG *n* (%)	TDF/FTC/EFV *n* (%)	*p* - *χ*^2^
BMI > 30			
Baseline	21/310 (7%)	25/303 (8%)	0.51
Week 192	69/255 (27%)	37/228 (16%)	0.01
SBP ≥ 130 or DBP ≥ 85			
Baseline	61/310 (20%)	62/303 (20%)	0.94
Week 192	111/255 (44%)	72/228 (32%)	0.01
Fasting glucose ≥5.6			
Baseline	17/310 (5%)	16/303 (5%)	0.97
Week 192	47/255 (18%)	37/228 (16%)	0.61
Triglycerides ≥1.7			
Baseline	32/310 (10%)	20/303 (7%)	0.12
Week 192	19/255 (7%)	19/228 (8%)	0.85
HDL < 1.29			
Baseline	205/310 (66%)	197/303 (65%)	0.71
Week 192	107/255 (42%)	70/228 (31%)	0.01

### Metabolic syndrome

In the NAMSAL trial, at baseline 18 (3%) participants had metabolic syndrome with no difference between the two treatment arms. Across all participants, the incidence of treatment-emergent metabolic syndrome was 14% (TDF/3TC + DTG) and 5% (TDF/3TC + EFV) at any time up to week 192 (*p* < 0.01) (Table 4B). The incidence of treatment-emergent metabolic syndrome was significantly greater in the TDF/3TC/DTG arm compared to the TDF/3TC/EFV arm in all patients (*p* < 0.001), and in males (*p* = 0.002) up to week 192 of the trial. The differences were not significant in females.

**Table 4B:** Treatment emergent metabolic syndrome at any time point: NAMSAL.

	TDF/FTC + DTG *n* (%)	TDF/FTC/EFV *n* (%)	*p* - *χ*^2^
All participants	41/287 (14%)	14/271 (5%)	0.00
Female	19/182 (10%)	13/185 (7%)	0.33
Male	22/105 (21%)	1/86 (1%)	0.00

## Discussion

In ADVANCE, across all study visits to week 192, treatment-emergent metabolic syndrome was present in 15% (TAF/FTC + DTG), 10% (TDF/FTC + DTG) and 7% (TDF/FTC/EFV) of participants. In NAMSAL the incidence of treatment-emergent metabolic syndrome was 14% (TDF/3TC + DTG) and 5% (TDF/3TC + EFV) at any time up to week 192. We highlight treatment-emergent metabolic syndrome associated with dolutegravir, likely driven by obesity. There is some evidence of metabolic syndrome associated with INSTI use in other trials. However, there is limited data coming from other randomised trials.

In a Zambian cross-sectional study, a DTG-based regimen was associated with metabolic syndrome (OR: 2.10, 95% CI: 1.05–4.20) (19). The ACTG A5001 and A5322 trials observed changes in weight gain to correspond with lower levels of HDL, and higher levels of LDL cholesterol, total cholesterol, triglyceride levels and fasting glucose (20). Similarly, 10% weight gain in NAMSAL was associated with a rise in cholesterol levels (21).

By contrast, in the REPRIEVE cohort study, despite being associated with a significant higher odds of developing obesity, INSTI use was not associated with metabolic syndrome, or differences in glucose, LDL, or hypertension (22). A randomised switch study from TDF/FTC/NNRTI to ABC/3TC/DTG saw significant weight gain in the DTG arm, but no associated reduced insulin sensitivity or treatment-emergent metabolic syndrome (23). Similarly, in the ATHENA cohort, weight gain of ≥10% was observed following switch to INSTI and/or TAF, without significant changes in metabolic parameters (24). In the TANGO trial, switching from a 3-/4-drug TAF based regimen to DTG/3TC, resulted in improvements in metabolic parameters (25). No significant differences in odds of metabolic syndrome were seen between the treatment groups in this trial (25).

Case reports have described incidences of hyperglycaemia associated with INSTI use (26, 27, 28, 29). In some of these cases, the hyperglycaemia was independent of weight gain, and associated with ketoacidosis, suggestive that the mechanism of dysglycaemia may not be associated with obesity. Another study showed cases of hyperglycaemia in pre-treated patients independent of weight gain and associated with ketoacidosis (30). There is mixed evidence relating INSTIs to insulin resistance. Some studies have shown association with insulin resistance (31, 32) and small rises in glycated haemoglobin (33, 34). A recent analysis of the FDA Adverse Event Reporting System linked INSTIs to greater odds of hyperglycaemia or new onset diabetes (ROR: 2.16, CI: 1.96–2.38) (35). However, there is also evidence opposing the association of INSTIs to insulin resistance and diabetes (36, 37).

Previous analyses of the ADVANCE trial looking at 10-year risk of developing diabetes using the QDiabetes equation, a risk equation validated in populations of Black ethnicity, have shown that TAF/FTC + DTG was associated with greater risk of developing diabetes (38).

INSTIs have been associated with increases in fat gain and body circumference (39, 40, 41, 42, 43). In the ADVANCE trial, DTG, combined with TAF was associated with increases in both mass and volume of VAT compared with the TDF/FTC/DTG and the TDF/FTC + EFV arms (16). They have generally been found to be lipid neutral (44, 45, 46, 47).

In two observational studies, dolutegravir was not associated with a significant change in glucose metabolism (48, 49). However, both studies were based on follow-up to 48 weeks. Such short-term studies may not be able to demonstrate metabolic syndrome.

### Strengths and limitations

A strength of this analysis is that both NAMSAL and ADVANCE were randomised controlled trials which included follow up data until week 192. Participants were recruited across different regions from HIV testing sites and hospitals which increases the generalisability of the analysis. A limitation is that in both trials, participants were lost to follow up to week 192.

### Implications for the future

This paper highlights new evidence of metabolic syndrome, primarily driven by weight gain and obesity, associated with the use of INSTIs. There is currently limited data from other randomised trials, particularly with INSTI use. Metabolic syndrome has a multitude of adverse health consequences which must be monitored for. Clinicians initiating or monitoring patients on INSTI-based ART must counsel for lifestyle optimisation to prevent these effects. Close monitoring of glucose, blood pressure and lipids is essential, with prompt initiation of anti-diabetic medications, anti-hypertensives, and anti-cholesterol agents where required. For those who have developed clinical obesity, anti-obesity drugs should also be considered to enhance weight loss. These can be cheaply manufactured and serve as an alternative to bariatric surgery which may not be a feasible option in low- to middle-income countries.

## Data Availability

Data can be made available if needed. Requests to access these datasets should be directed to Manya Mirchandani manya.6269@gmail.com.
